# Preference‐based patient participation for most, if not all: A cross‐sectional study of patient participation amongst persons with end‐stage kidney disease

**DOI:** 10.1111/hex.13323

**Published:** 2021-08-01

**Authors:** Caroline Martinsson, Fredrik Uhlin, Marika Wenemark, Ann Catrine Eldh

**Affiliations:** ^1^ Department of Health, Medicine and Caring Sciences, Faculty of Medicine and Health Sciences Linköping University Linköping Sweden; ^2^ Department of Nephrology Region Östergötland Linköping Sweden; ^3^ Department of Health Technologies Tallinn University of Technology (TalTech) Tallinn Estonia; ^4^ Unit of Public Health and Statistics Region Östergötland Linköping Sweden; ^5^ Department of Public Health and Caring Sciences Uppsala University Uppsala Sweden

**Keywords:** engagement, involvement, patient‐centred care, patient participation, person‐centred care, questionnaire

## Abstract

**Background:**

Patient participation is considered central for good healthcare. Yet, the concept is not fully understood when it comes to patients' experiences of participation in conjunction with their preferences, particularly in long‐term healthcare. The aim of this study was to investigate the extent and variation of preference‐based patient participation in patients with end‐stage kidney disease (ESKD).

**Methods:**

A cross‐sectional study was conducted with 346 patients in renal care. The main variables were patients' preferences for and experiences of patient participation, determined using the Patient Preferences for Patient Participation tool, the 4Ps. Analyses identified the degree of match between preferences and experiences, that is, the preference‐based patient participation measure.

**Results:**

Overall, 57%–84% of the patients reached a sufficient level of preference‐based patient participation on the items, while 2%–12% reached an insufficient level. A mismatch indicated either less or more participation than preferred; for example, 40% had less experience than preferred for taking part in planning, and 40% had more than preferred for managing treatment.

**Conclusion:**

This study shows that, although many patients reach a sufficient level of preference‐based patient participation, this is not the case for all patients and/or attributes. Further opportunities for a mutual understanding of patients' preferences are needed for healthcare professionals to support person‐centred patient participation.

**Patient or Public Contribution:**

The 4Ps is manufactured in collaboration with people with experience of the patient role, and persons living with ESKD were engaged in identifying their preferences and experiences of participation in renal care.

## 1 INTRODUCTION

Worldwide, healthcare legislation and policies incorporate patients' rights to influence their own health, healthcare and treatment—often defined as ‘patient participation’.^1^, ^2^ Patient participation is known to have positive outcomes for health^3^: Participation may enhance empowerment and satisfaction with care,^2^ as well as independence^4^ and self‐care management.^5^ However, patient participation is a comprehensive concept,^5^, ^6^ signifying for example being involved in a life situation*,* ^7^ as well as having a narrower meaning related to taking part in decision‐making about healthcare issues.^3^ According to concept analyses, patient participation entails both particular activities and intellectual features, such as managing self‐care or experiencing conditions for mutual communication.^5^, ^8^, ^9^, ^10^, ^11^ In addition, patients conceptualise participation in a broad sense, comprising for example the sharing of information and knowledge with healthcare professionals and fellow patients. Furthermore, to patients, participation connotes partaking in planning and managing self‐care, indicating that patient participation comprises engagement around the clock and not just during a healthcare encounter.^12^

Despite the lack of a single definition, the World Health Organization claims that, to improve health and well‐being, any person in the role of a patient should have the option to engage in their care and treatment in accordance with their own preferences.^1^ Consequently, any appraisal of patient participation in healthcare requires measures for patients to define their preferences for patient participation.

One group of people who need to engage in health‐related issues are individuals living with end‐stage kidney disease (ESKD). This long‐term condition imposes repeated and/or prolonged contact with healthcare and often involves physical symptoms.^13^ Fatigue is a common side effect of both the ESKD and the dialysis treatment, if necessary,^14^ indicating that both the illness and the treatment may also impact upon one's everyday life,^15^ requiring engagement around the clock.^12^, ^16^ Globally, there are approximately 700 million people with ESKD,^17^ and approximately two million people receive renal replacement therapy (RRT) with dialysis.^18^ Both men and women are affected, although a majority are men.^19^ The population with ESKD and RRT in Sweden consists of roughly 10,000 individuals, most of them aged 60 years or older.^20^

However, patient engagement in renal care has attracted limited attention. Although Årestedt et al.^21^ found that, in dialysis care, lack of a common conceptualisation of patient participation between staff and patients may hinder patient participation, Aasen et al.^22^ found that people undergoing dialysis experience shortcomings related to being involved in their treatment.

Measures to conceptualise participation from the patient perspective are scarce,^11^ but the Patient Preferences for Patient Participation (4Ps) tool incorporates what people with experience of the patient role define as patient participation.^23^, ^24^ The 4Ps tool assembles 12 items that represent both a perspective on ‘sharing of’ (as in experiences, knowledge and information) and ‘sharing in’ (healthcare plans, goals and decisions and self‐care management),^25^ consistent with the origin of participation as in ‘to share’.^26^ The 4Ps tool captures both patients' preferences for and their experiences of patient participation,^23^, ^27^, ^28^ making it possible to measure preference‐based patient participation, that is, the extent to which patients' experiences of participation match their preferences. Preference‐based patient participation is intended as a measure for person‐centred care, by which staff can provide conditions for the patient to take part in health‐related matters according to his or her preferences for participation.^25^

To date, few studies have addressed the extent to which patients' experiences of patient participation align with their preferences. The perspectives may vary between patients and staff as to what patient participation is.^21^ The aim of this study was to investigate the extent and variation of preference‐based patient participation among patients with ESKD.

## 2 METHODS

### 2.1 Study design

A cross‐sectional study was conducted.

### 2.2 Setting

Out of 11 eligible renal care sites in the southeast of Sweden, 9 agreed to take part by means of informed consent provided by each head of clinic. The nine sites were located across different types of hospitals (county, regional and university hospitals); all nine had dialysis units and seven also had outpatient care units.

### 2.3 Participants

Patients were enlisted by a consecutive sampling method. The criteria for inclusion were as follows: aged 18 years or older, and diagnosed with ESKD stage IV (estimated glomerular filtration rate [eGRF] 15–29 ml/min) or stage V (eGRF < 15 ml/min).^17^ Furthermore, participants needed to be able to communicate in Swedish without an interpreter. The sole exclusion criterion was a recognised cognitive impairment.

### 2.4 Data collection procedures

After agreement was secured for the sites, a contact person at each unit (either a first‐line manager or an assigned nurse) identified patients according to the above criteria. All contact persons were asked to report to the first author how many patients were eligible for the study in terms of the inclusion/exclusion criteria. The contact person could choose to either: (a) provide details regarding eligible patients to the first author, who sent information about the study to the patients via regular mail or (b) distribute the information to potential study participants himself or herself. Hence, all potential study participants received written information about the study, along with the 4Ps tool, and a short questionnaire covering demographics (including age, sex and type and duration of healthcare contact for renal care). In addition, this package included a slip to register consent, and a prepaid reply envelope. If they agreed to participate, patients were instructed to send the informed consent along with the completed 4Ps tool and demographic questionnaire to the first author. If they wanted to decline, it was suggested that they return the empty reply envelope to avoid reminders. Nonrespondents were sent two reminders at 3–4‐week intervals to their home addresses, whereas in the three units where dissemination was conducted by a contact person, he or she was asked to nudge all the patients who had been invited with a more general, verbal reminder. All patients sent their responses to the first author, and thus no information was shared with the sites as to which patients had consented or declined to participate, or their answers to the 4Ps or the short questionnaire. All participants were asked to report their contemporary preferences for and experiences of patient participation, considering their current healthcare contact with the renal unit.

A total of 729 patients received the study information package, 633 distributed by the first author and 96 by the contact persons. However, a full account of how many patients were asked at the three sites where contact persons distributed the information package are not known. Altogether, 363 patients consented (in writing) to participate, and 346 (47%) completed the 4Ps. Data were collected between August and December 2019.

### 2.5 The research tool: The 4Ps

**Figure 1:**
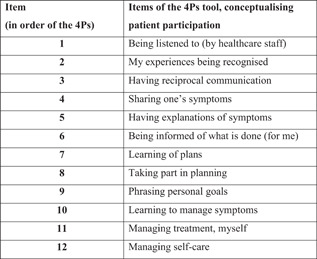
The 12 items conceptualising participation, as set out in the Patient Preferences for Patient Participation (4Ps) tool^23^, ^24^

The 4Ps incorporates two sections, both using the same 12 items conceptualising participation (Figure 1).^23^, ^24^ In section 1, the respondent is instructed to indicate his or her preferences for patient participation by means of each item and the response alternatives ‘unimportant’, ‘somewhat important’, ‘very important’ or ‘crucial’ (for my participation, as a patient). In section 2, the patient indicates his or her experience of patient participation, marking each item with one of the response alternatives (I have experienced patient participation) ‘not at all’, ‘to some extent’, ‘to a large extent’ or ‘entirely’. Previous evaluations of the 4Ps' psychometric properties imply reasonable validity and reliability.^24^, ^29^

### 2.6 Data analysis

The responses to the 4Ps and the demographic data were registered and analysed in the software *IBM Statistical Package for Social Sciences* (SPSS) version 26.

Descriptive statistics of participants' characteristics were summarised and presented as frequencies, means (*m*) and range. Comparison of means regarding variables such as age were calculated using Student's *t *test.

**Figure 2:**
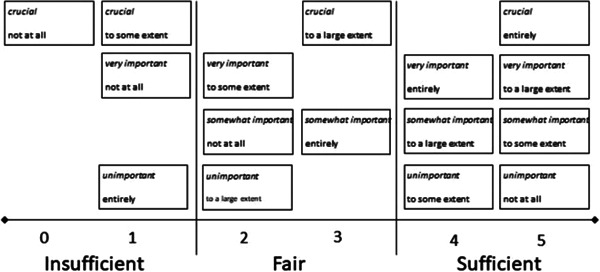
Ranks and levels for the match and mismatch between patient preferences (italicised text) and experiences of patient participation (roman text). Originally published by Eldh et al.^28^

Data representing preferences for and experiences of patient participation were initially analysed separately, comprising each of the 12 items in the two sections. Subsequently, the two sections were analysed jointly: For each patient, a preference‐based participation score for each item was identified, signifying the degree of match or mismatch between the patient's preferences and experiences. With four fixed‐response alternatives in each of the two sections, there are all in all 16 possible combinations vis‐à‐vis preference‐based patient participation, that is, degree of match between self‐reported preference and experience for patient participation. These 16 combinations are ordered from 0 to 5 and then classified into three levels: insufficient (rank 0–1), fair (2–3) and sufficient (4–5) preference‐based patient participation,^28^ as presented in Figure 2. The levels operate a general assumption that the closer the match between a patient's preferences and experiences, the better it represents ideal conditions for preference‐based patient participation. Yet, if there is no complete match between the patient's preferences and experiences, it is better if the experiences exceed the patient's preferences, that is, having further opportunities to participate, than the opposite (i.e., if experiences are lesser than one's preferences). These assumptions build on economic and ethical theories and have transpired as conditions for patient participation and nonparticipation, respectively.^30^, ^31^ Since preference‐based patient participation is expressed in ordinal variables, the *χ* ^2^ test and Fisher's exact test were used to study differences between subgroups (such as predialysis/dialysis, men/women). A *p* < .05 was used to distinguish statistical significance.

## 3 RESULTS

This study investigated the extent and variation of preference‐based patient participation within the context of renal care. The findings reported are in the following order: A description of participants' characteristics, the degree of match between the ESKD patients' experiences and preferences, that is, the preference‐based patient participation measure, and the variation in preference‐based patient participation. Supplementary files 1 and 2 provide the details of the patients' preferences for and experiences of participation, respectively.

### 3.1 Characteristics of participants

The 346 patients responding to the 4Ps were aged between 27 and 93 years (mean age: 70 years). Most participants had more than three years' experience of being patients in renal care; half were patients in a predialysis context, that is, they had outpatient contact a couple of times a year, and the other half were in RRT, undergoing regular dialysis 3–4 times a week (delivered in outpatient settings). The most common forms of RRT were haemodialysis (64%, *n* = 119) and peritoneal dialysis (9%, *n* = 17). The patients' RRT had most often been underway for ≤2 years (36%, *n* = 68), followed by 3–5 years (30%, *n* = 56), while 17% (*n* = 32) had been in RRT for 6 years or longer. The full details of age, sex and years of experience of renal care for all participants are provided in Table 1.

**Table 1:** Demographic characteristics of the study participants

		Type of ESKD contact	
	All, % (*n*)	Predialysis, % (*n*)	Dialysis, % (*n*)
	**346**	**159**	**187**
Men	63 (219)	64 (101)	63 (118)
Women	37 (127)	36 (58)	37 (69)
Age, mean ± *SD* (range)	70 ± 12, 73 (27–93)	71 ± 12, 73 (27–88)	70 ± 13, 73 (28–93)
Years with experience of renal care			
≤2 years	9 (27)	8 (11)	9 (15)
3–5 years	33 (102)	32 (45)	34 (56)
≥6 years	56 (172)	57 (80)	55 (90)

Abbreviation: ESKD, end‐stage kidney disease.

### 3.2 Preference‐based patient participation

Comparing the patients' experiences of patient participation with their preferences for participation, a concordance was often noted. Thus, the experience of a particular attribute for patient participation completely matched the patient's preference for that item, indicating that, where an attribute was considered crucial for participation, this was matched with an experience of having ‘entirely’ experienced conditions for that attribute, or if the patient considered an attribute ‘unimportant’ for his or her participation, he or she had experienced no conditions for the same item. While many patients' data showed a complete match, there were also degrees of mismatch between patients' experiences of and preferences for participation. Some measures indicated a complete mismatch: for example, in cases where an item was considered crucial for participation, but the patient had experienced no conditions at all for participation in that matter. Overall, there was some variation as to which attributes of patient participation achieved a complete match and which displayed a less favourable coherence between preferences for and experiences of patient participation.

**Figure 3:**
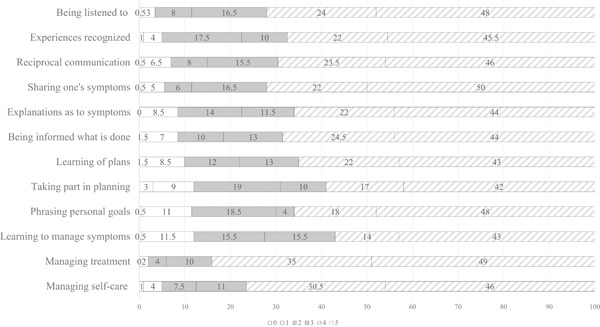
Ranks and levels of preference‐based patient participation per item (%): Ranks 0–1 equate to level insufficient; ranks 2–3 equate to level fair; and ranks 4–5 equate to level sufficient

When studying ranks 4–5, meaning sufficient preference‐based patient participation, approximately two‐thirds reached a sufficient level of preference‐based participation. This was noted especially in terms of ‘managing treatment myself’ (84%, *n* = 289) and ‘performing self‐care’ (76,5% *n* = 266), while the most mismatches (i.e., rank 0) were for participation, as in ‘taking part in planning’ (3%, *n* = 9). All outcome ranks (0–5, representing no to complete match) and levels (from left to right: Insufficient, fair and sufficient preference‐based participation) are reported in Figure 3 for each item.

The variations represented differences in terms of type of renal care: More individuals undergoing treatment with dialysis had preferences and experiences that matched a sufficient level of preference‐based participation, compared with individuals in predialysis care. A significant difference was detected regarding *learning what is planned for me* (*p* = .001), *having conditions to take part in planning of care/treatment* (*p* = .03) and *having opportunities to phrase my own goals* (*p* = .045).

**Figure 4:**
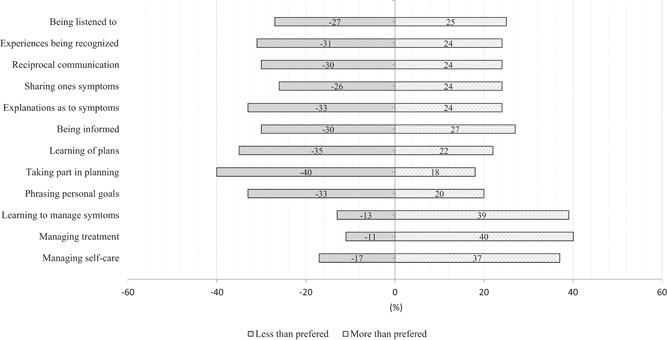
Proportion of matching between the patients' experiences and preferences, indicating whether conditions for patient participation were less or more than preferred

A mismatch between experiences and preferences can represent either a lower experience than one's preference for patient participation, or the experience exceeding one's preference (for a particular attribute of participation). In this case, items corresponding to participation in self‐care management more often conveyed experiences exceeding the patients' preferences. In addition, 40% of the responses had a mismatch between experiences and preferences in terms of taking part in planning, with lesser experiences than the patients' preferences. All mismatches in terms of less or more participation than preferred, respectively, are provided in Figure 4, indicating that, for nine items, the mismatch more often represented preferences not being met.

## 4 DISCUSSION

Legislation and policies suggest that patient participation should align with the principles of person‐centred care, that is, that a patient's needs and condition should guide the provision of care, including opportunities for participation. Considering our findings, this is often, but not always, the case for patients with ESKD in renal care. Moreover, some properties of patient participation are seemingly more promoted than others. Hence, we suggest a discussion about which elements represent a match and mismatch, respectively, and for whom. In addition, we address the need to assess patient participation from a patient perspective, thus comprising patients' preferences for participation. Lastly, we discuss what is at stake in terms of person‐centred care if healthcare services fail to provide for preference‐based patient participation.

Preference‐based participation is a measure intended to distinguish what a patient wishes with regard to his or her participation and what he or she finds in the healthcare interaction. In this cross‐sectional study, the 4Ps research tool was used to measure and demonstrate preference‐based patient participation. The findings show a high overall degree of match between patients' preferences and experiences in terms of patient participation, especially for items such as being listened to by professionals and/or managing one's treatment. Supposedly, this indicates that the patients' engagement aligns with the conditions for participation that they, as individuals, favour.^28^ This could indicate that each individual's varying needs are recognised and acknowledged by the healthcare services.^2^

Previous studies have shown that participation occurs when one as a patient has opportunities for dialogue, including a mutual sharing of experiences and knowledge.^4^, ^5^ This represents a sharing of the professional's expertise, but also sharing one's lived experience and understanding of one's body and symptoms as a patient.^11^ Living with a long‐term condition, such as ESKD, is often associated with acquiring extensive recognition of the bodily manifestations of the illness.^32^ However, patients may find it difficult to share such experiences,^4^, ^33^ particularly if they are not requested or considered appropriate, for example a third of the study participants had experienced less conditions than preferred to partake in terms of sharing their symptoms or having explanations to their symptoms (Figure 4). Rather, a mutual sense of trust and engagement is needed to build confidence in discussing such issues.^10^, ^34^ Such relations are more easily built over the long term, through recurring contact with the same professionals.^35^

Although this study suggests that typically there is a match between patients' experiences of participation and their preferences, some of the attributes of patient participation indicate more of a mismatch than others, for example, with preferences higher than the patients' actual experience and vice versa. One difference was noted between patients in RRT and people in a predialysis phase. For patients in dialysis care, participation has been found to encompass a variety of attributes, including the sharing of experiences (as in reciprocal communication)^3^, ^11^ and health and healthcare activities.^12^ Thus, some patients would evidently prefer to participate more in the latter sense, recognising their capacity to engage in the planning of care and treatment. Patients in RRT have often lived with ESKD for long, and while RRT may require haemodialysis three or four times a week,^36^ these patients can have had regular contact with renal care^20^ as well as with a limited number of nursing staff for a long time. ESKD patients in predialysis, on the other hand, may only visit an outpatient unit a couple of times per year, interacting with different healthcare professionals in these visits. The repeated and high frequency of care contacts associated with RRT can facilitate participation by means of continuity in care relations, providing opportunities for the mutual exchange of knowledge and experience.^21^ Yet, this requires a recognition of the establishment of collaboration, for example by nurse‐led outpatient care units,^37^, ^38^ but, most importantly, recognition of the individual patient's needs.^37^

Healthcare legislation and policies prescribe that healthcare professionals must involve patients in their care, on the patient's terms,^1^, ^2^ although healthcare professionals have been found to overestimate patients' readiness to enact healthcare.^39^ Staff in dialysis care may favour engaging patients in the sense of performing part of or the full dialysis^12^, ^40^ as this has been found to reinforce a sense of control and thus enhance the individual's well‐being.^41^ Yet, ESKD has a slow yet declining progression,^20^ and one size does not fit all: Living with this condition might challenge one's self‐esteem and hamper long‐term engagement. Gender,^42^ age,^43^, ^44^ current health status^45^, ^46^, ^47^ and perceived capability^48^ may also affect how a patient prefers to participate. A lack of motivation has been found to lead to less engagement outside the healthcare encounter,^49^ calling for a further, mutual dialogue when planning self‐care activities. Our findings suggest that ESKD patients may wish for engagement in setting goals and engaging in plans, calling for further opportunities to initiate such joint ventures.^3^ Supposedly, professionals need better support to recognise the beliefs and preferences of the individual patient, and thus to enable the route to participation that the patient prefers.^2^, ^28^, ^50^

Our results suggest that the overall conditions for person‐centred participation do occur in the ESKD care context, but potentially intermittently and unpredictably. Failure to recognise a patient's preferences and experiences can cause unnecessary struggle for the individual,^51^ but the mounting opportunities for preference‐based patient participation should be available for everyone.^25^, ^52^ Without knowing a patient's preferences, person‐centred participation, where beliefs and values are emphasised, cannot be fully achieved.^48^ It may also hamper the opportunities for patients to engage on their own terms^49^, ^53^ and thus their prospects of managing their health in everyday life.^49^, ^51^ The 4Ps provides a potential tool to enable the recognition of patient preferences,^29^ although the transition from measuring preference‐based patient participation to engaging staff and patients in implementing such a tool^54^, ^55^ will probably include changing routines. Thus, while the 4Ps may serve preference‐based patient participation, further studies are needed to investigate whether and how it can also serve the quality of care in everyday practice.^56^

### 4.1 Strengths and limitations

This study includes one region of Sweden only, although it does include typical public hospitals (regional, university and local hospitals) serving large catchment areas across rural and urban districts, including towns and cities. Thus, the study was performed within a context similar to the general Swedish healthcare context,^20^ and the demographics of the participants are similar to the larger subgroup of people living with ESKD. This makes the findings potentially transferable to similar healthcare contexts.

The study used the 4Ps tool to address patients' preferences and experiences of participation: The respondents were asked to consider their preferences for and experiences of patient participation at one point of time, and to consider their current healthcare contact. Thus, findings represent a single data‐collection point. The 4Ps captures and exhausts the concept of patient participation, is easy to use and serves as a means to put forward one's preferences and experiences^23^, ^24^; in this study, a layout where the patient reports his or her preferences for and experiences of participation simultaneously was used. For patients with a less recent visit to an outpatient service, their preference‐based participation may have been influenced by hindsight. Yet, since aspects of significance tend to linger, as do experiences of impact on one's life (like health issues),^57^ even a memory of a previous healthcare interaction can represent patients' current perception of patient participation. However, further studies could use measurements of the 4Ps on different occasions (with the same patients) to better understand if and how preferences and experiences change over time.

Because the study lacks some recruitment feedback (from three sites), there is no full account of how many patients: (a) were eligible to take part in the study and/or (b) were informed and declined to participate. Although the sample of participants is fairly representative, the mean age of the study participants was somewhat higher than the general ESKD population. Furthermore, the response rate may have been influenced by whether the initial query was made via mail or by a local contact person. However, full confidentiality was ensured because no reports regarding consent or decline were given to the sites. Even where the information package and questionnaires were handed over to the patient by the healthcare contact, all further correspondence was with the research team only.

## 5 CONCLUSIONS

Patients with ESKD most often reached a good match between their preferences for and experiences of patient participation in renal care. However, for all items of patient participation, there was some degree of mismatch, indicating that a proportion of patients are not able to participate to the extent or in the way that they want. Furthermore, certain aspects of participation are more often met than others in terms of preference‐based patient participation, such as managing treatment and self‐care. Previous studies have suggested that there is a lack of coherence between healthcare professionals' and patients' conceptualisations of patient participation, which could explain why not all patients are provided with conditions that match their preferences for participation. Thus, further studies are needed to better understand how to assess preference‐based patient participation.

## 6 IMPLICATIONS FOR PRACTICE

While patients in renal care are largely able to participate in their own care to the extent and in the ways that they prefer, there are attributes of participation that are less in line with the patients' preferences. The match between patients' preferences for and experiences of patient participation may be unintentional or deliberate, although the evident mismatch for a number of attributes known to conceptualise patient participation indicates the former. Thus, further dialogues between healthcare professionals and patients about their preferences for participation are suggested. To provide for patient participation in accordance with a person‐centred norm, opportunities for patients to share their preferences and experiences of participation are required for example, by a clinical application of the 4Ps or similar communication means.

## 7 CONFLICT OF INTERESTS

The authors declare that there are no conflicts of interest. The 4Ps is protected by copyright but available free of charge by agreement with the last author.

## AUTHOR CONTRIBUTIONS

Ann Catrine Eldh and Fredrik Uhlin designed the study. Caroline Martinsson collected the data and performed the analyses, in dialogue with Ann Catrine Eldh and Fredrik Uhlin and guided by Marika Wenemark. Caroline Martinsson drafted the manuscript in collaboration with Ann Catrine Eldh; Fredrik Uhlin and Marika Wenemark made substantial contributions by means of revising and amending the text. All authors have approved the final version of the manuscript.

## ETHICS STATEMENT

Approval was obtained from the Swedish Ethical Review Authority (registration no. 2019‐02748).

## Supporting information

Supporting information.

Supporting information.

## Data Availability

Data are available on request from the authors.
