# Supplementary material for: Preference‐based patient participation for most, if not all: A cross‐sectional study of patient participation amongst persons with end‐stage kidney disease
Source: Health Expect. 2021 Aug 1;24(5):1833–41. doi: 10.1111/hex.13323 (PMC8483194; doi:10.1111/hex.13323)

**SUPPLEMENTARY FILE**

The figure below represents patients' preferences for patient participation among the study participants. Illustrated as frequencies (%) per response alternatives for each item of the Patient Preferences for Patient Participation tool, the 4Ps.

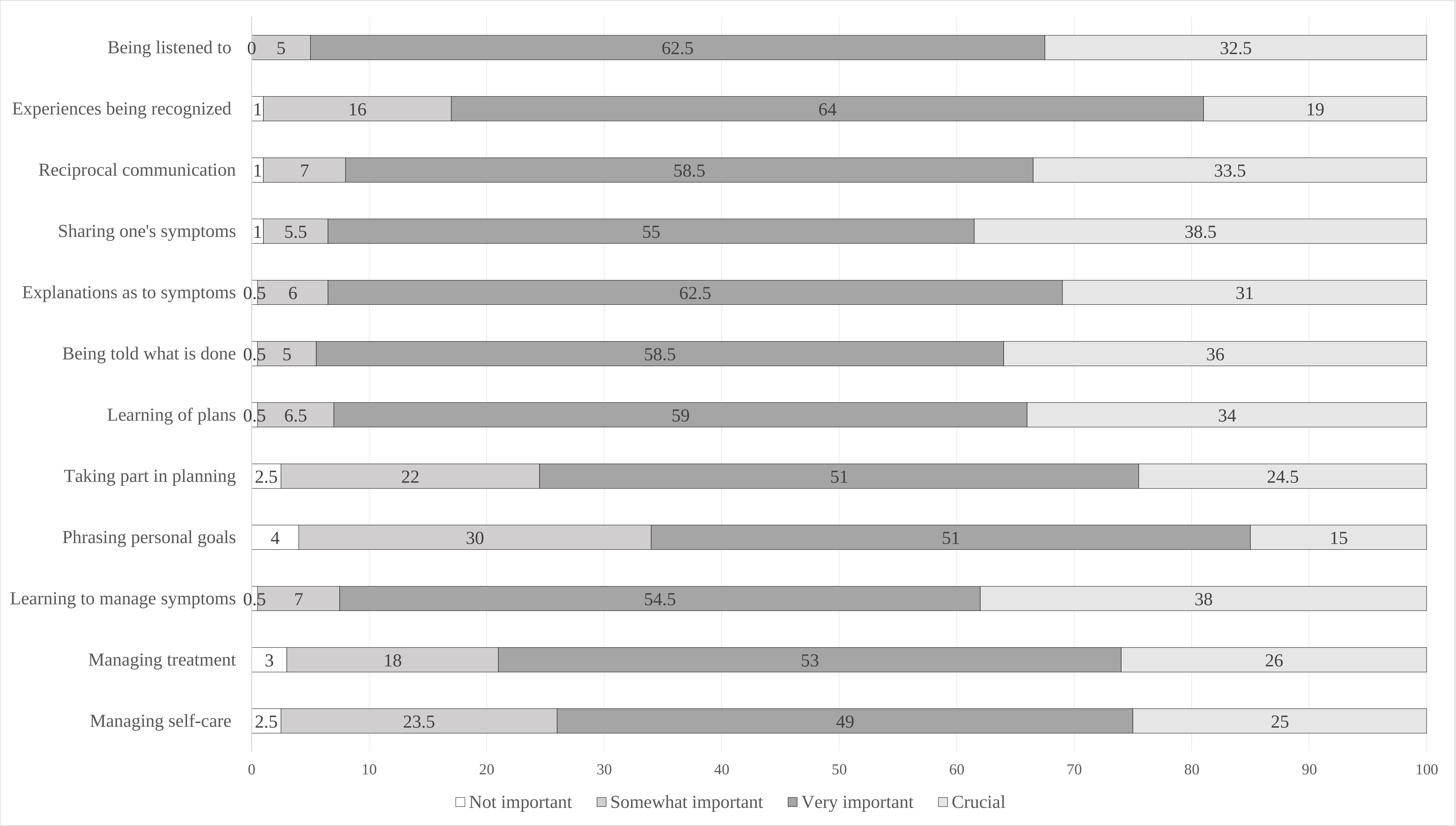


.

The figure below represents patients' experiences for patient participation among the study participants. Illustrated as frequencies (%) per response alternatives for each item of the Patient Preferences for Patient Participation tool, the 4Ps.

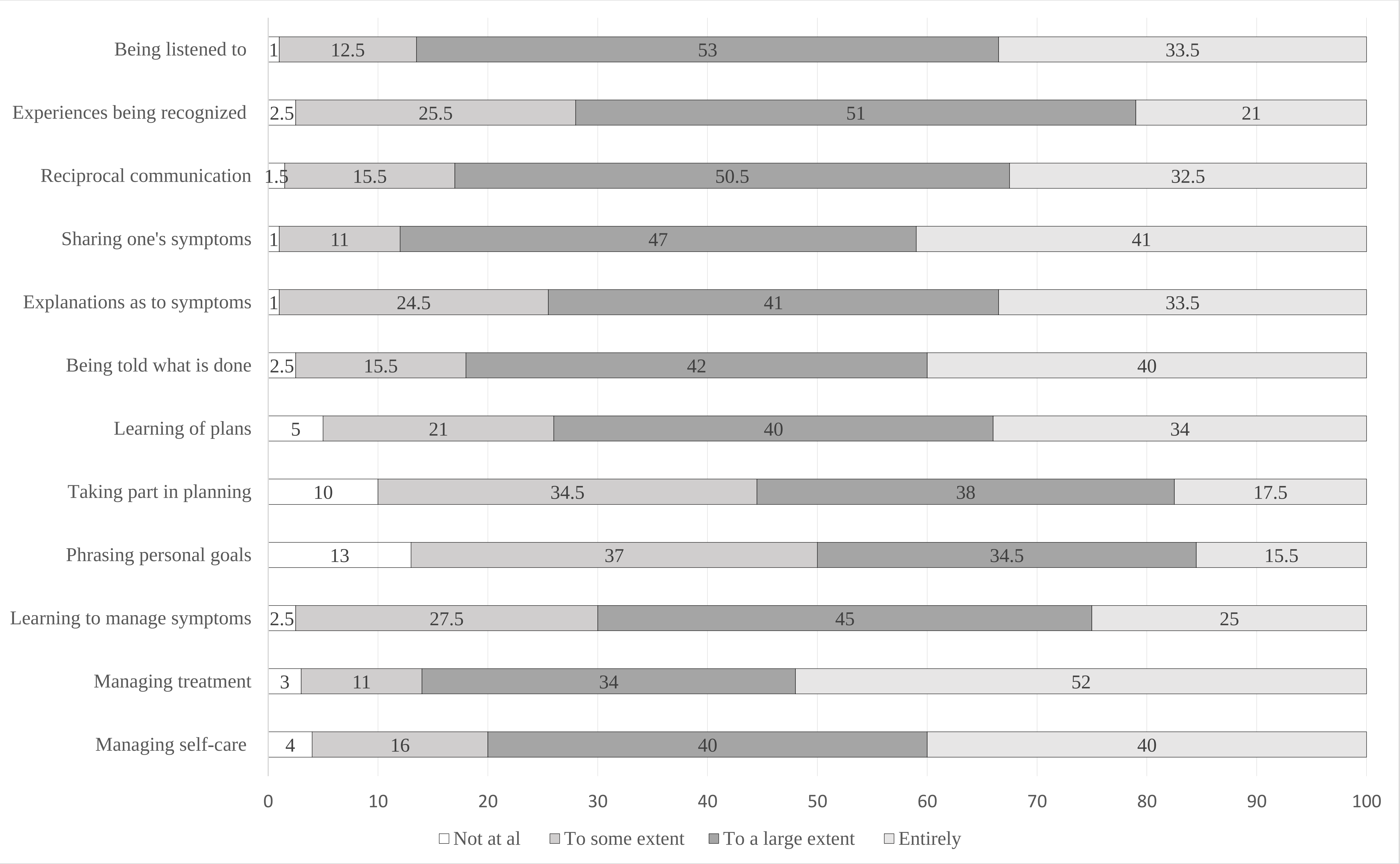

Supplement: Supplementary file 2 — Supporting information. [file HEX-24-1833-s001.pdf]
